# Hospitals and Insured Persons' Fees

**Published:** 1921-03-12

**Authors:** 


### The Hospital

The Workers' Newspaper of Administrative Medicine and Institutional Life, Administration, National Insurance and Health.

No. 1814, Vol. LXIX. SATURDAY, MARCH 12, 1921. PRICE TWOPENCE

### HOSPITALS AND INSURED PERSONS' FEES.

We cannot attach to the report of the Quarterly Court of the London Hospital the same dramatic construction, in respect of payments to be made by National Health Insurance patients, as has been read into Lord Knutsford's speech by some other people. Possibly this is so because we appreciate his peculiar faculty of being misunderstood by the press, and we examine any statement made by him with extra care. As he says, he was not responsible for the heading, "Ultimatum from the Hospitals," with which his announcement has been labelled.

What we understand to be the case is that the London Hospital is spending something like £70,000 a year on insured patients, and is compelled to search for an alternative should the Approved Societies continue to neglect their responsibilities. The position at the London is not a new one, but is simply a large version of that of other voluntary hospitals; at the Royal Infirmary, Liverpool, for instance, 25 per cent. of the patients are insured, and if proper attention were given to what is described as a glaring grievance the hospital ought reasonably to hope to receive from £3,000 to £4,000 a year. It is a state of affairs which must have been brought to the notice of Lord Cave's Committee by witness after witness, and one which cannot continue without considerable amelioration. The Approved Societies owe the hospitals a debt of honour which they are now in a position to pay. There can be no doubt that the Societies would have had to pay much larger sums in "sickness benefit," as Lord Knutsford points out, if the voluntary hospitals had not helped their members to get well more quickly than they could possibly have done without such help. In other words, the savings made by the Societies have been made at the expense of the voluntary hospitals, which "are practically financing involuntarily Approved Societies."

The Ministry of Health has made a further announcement on the question of the valuation of Approved Societies that is now proceeding, but has not yet been completed. The results are sufficient to show that a very considerable sum remains in the hands of the Societies for distribution to members in the form of additional benefits. The surplus already disclosed amounts to £3,600,000, or over £1 6s. per insured person. It will be remembered that in the original Act the voluntary hospitals were passed over with practically no recognition, although it was clear to most people that the Act would be unworkable without their aid. Not only was there no "hospital benefit," but the sickness benefit of insured persons ceased upon their admission to a hospital, although the Societies were given permissive powers to hand over all or part of the benefit, with the concurrence of the patient, to the institution. How seldom this occurred is known to hospital secretaries. Quite recently, the Health Ministry circular informs us, a regulation has been made allowing the surplus in Societies to be devoted in whole or in part towards the maintenance and treatment of members in hospitals and convalescent homes. It is anticipated, says the same authority, "especially in view of the present financial necessities of the hospitals," that quite a considerable number of Societies will make arrangements for the institutional treatment of their members; moreover, Dr. Addison is aware that some of the largest Societies are contemplating this step in preference to an increase in the cash benefits.

We much resent the reference to present financial necessities. The only issue is that the hospitals have a clear claim which should be quickly satisfied quite irrespective of their financial necessities. When such a high authority as the Minister of Health introduces an element of pity into his examination of the claim of the hospitals it is not surprising that lesser officials, such as Sir Thomas Neill, of the National Amalgamated Approved Society, and Mr. P. Rockliff, of the Joint Committee of Approved Societies, adopt a rather ungracious tone, although in justice it must be conceded that they may have been misled by the construction of the ultimatum that has been given by some newspapers to Lord Knutsford's remarks. Both these gentlemen take the line that most insured persons already contribute to hospitals by means of the Hospital Saturday Fund or through workmen's collections. Far be it from us to depreciate what is being done by these sources of support, which, comparing London with the Provinces, are as yet in the first stages of development, and do not, with all the undoubted goodwill of the individual subscribers, yet touch the fringe of the situation. But both authorities admit, if with an unwilling air, that some consideration is due and some support should be given by members of the societies as a free-will offering.

We do not think that the London Hospital or any other Metropolitan hospital is out to exact its pound of flesh. Coercion of any kind is foreign to the spirit of the voluntary system. All that is desired is that every agency and individual in touch with hospitals must bear a share of the great task that these curative institutions have gradually assumed. It may be that the time has come to discontinue the use of generalities in making appeals to the generosity and the sense of justice of all, and that plain speaking, adapted to suit individual circumstances, is necessary. This and no more, we conceive, has come from the lips of one who may be described as a veteran in public-welfare service, and we thank the Chairman of the London Hospital for expressing the feelings of the majority of voluntary hospitals so plainly.

